# Permanent Contraction of the Fingers

**Published:** 1842-09

**Authors:** 


*Permanent Contraction of the Fingers.* By Prof. Velpeau.—Permanent flexion or contraction of the fingers depends on a great number of causes; it may arise from wounds, paralysis of the extensor muscles, anchylosis of the joints, tumors, shortening of the tendons, irregular cicatrices, &c. On the present occasion, however, I wish to direct your notice to that form which depends on contraction of the palmar fascia, or on the formation of fibrous bands extending from the palm of the hand to the fingers, and binding down the latter in their irregular position.

Before the remarks published in 1831, by Dupuytren, followed up by those of MM. Lemoine, Mandet, Vidal, Goyraud, &c. permanent contraction of the fingers had not been much noticed by surgeons. Boyer alludes to the affection under the name of *crispatura tendinum;* Sir Astley Cooper, also, speaks of permanent retraction of the fingers and toes, which he attributes to contraction of the sheaths of the tendons of the palmar and plantar aponeuroses. Dupuytren has clearly shown that the tendons are seldom engaged in this affection, but he has not so evidently demonstrated that it depends on induration, or shortening of one or more bands of the palmar fascia.

The disease now under consideration, is characterised by the formation of certain bands or chords, which are elevated beneath the skin, and extend along a considerable portion of the palmar surface of the finger; these bands generally occupy the median line, and are attached usually to the first phalanx, but sometimes prolonged to the second or third. According to Dupuytren this disease generally begins on the ring finger, and thence extends to the little finger; it increases gradually, without occasioning pain; the patient, at first, experiences a feeling of stiffness in the palm of the hand, and is unable to extend one or more fingers; the latter soon become contracted, and, in extreme cases, the tip of the finger rests on the palm of the hand. On examination, the first thing which we notice is a chord extending along the palm and finger; if you attempt to straighten the finger, this chord becomes more tense, and it disappears when you flex the finger completely; it is rounded off in shape, and forms a kind of bridge or prominence at the metacarpo phalangeol joint. The skin is wrinkled into arched folds, extending from the middle of the palm of the hand to the base of the finger. The disease, thus progressing, may attain its maximum degree of severity without causing any pain, or appearance of anchylosis; the joints are easily flexed, but cannot be extended by the most powerful efforts; Dupuytren has seen 150 pounds weight suspended on the bent finger without producing any effect on it.

This retraction of the fingers is a very disagreeable affection; it prevents the patient from seizing any large body, and, when he closes the fingers strongly, severe pain is felt. Persons laboring under permanent contraction of the fingers, are usually those who have made violent efforts with the palm of the hand, or been employed in occupations during which the palm and fingers are frequently pressed against hard bodies. Thus, it is met with amongst masons, ploughmen, watermen, coachmen, &c. Dupuytren mentions the case of a literary personage who was attacked in consequence of frequently using a round-handled office-seal for his letters.

What is the anatomical lesion which characterises permanent contraction of the fingers? Upon this point there exists a variety of opinions. Some attribute it to thickening and contraction of the skin; others, to spasm of the muscles; disease of the flexor tendons; inflammation and contraction of the sheaths of the tendons; some change in the joints and their lateral ligaments; finally, contraction of the palmar fascia. This latter idea was chiefly supported by Dupuytren and Sir Astley Cooper; the following case was the one which influenced Dupuytren's opinion:—An old man, who had been affected for many years with retraction of the fingers, died in the Hôtel-Dieu; Dupuytren examined the parts with great care; on removing the skin from the palm of the hand, it was found that the folds of integument completely disappeared, the palmar fascia was contracted and shortened, and several bands passed from its lower part to the sides of the deformed finger. On endeavoring to extend the fingers, Dupuytren perceived that the fascia and these bands were rendered more tense; he divided the bands, and effected, at once, complete extension of the contracted fingers; the tendons and their sheaths had not been touched; the joints likewise and bones were completely free from any appearance of alteration. From this case Dupuytren naturally concluded that the disease originated in the abnormal tension of the palmar fascia, a tension depending on contraction excited by the pressure of some hard substance on the palm of the hand. The case certainly shows that contraction of the fingers may sometimes depend on a change in the palmar fascia, but it does not prove that this is always the cause. The bands or hard chords, which, as I have already mentioned to you, are seen projecting under the skin, extend to the palmar surface of the first phalanx, and sometimes to the second or third phalanx. Now we know that the palmar fascia terminates at the root of the finger, by becoming attached to the sheaths of the tendons and ligaments; besides, the fascia does not cover the ball of the thumb or extend to its root, yet we have permanent contraction of the thumb, with the sub-cutaneous chords already noticed. Having determined, by dissection, that the palmar fascia remained sometimes intact, after the removal of these chords, I announced, in 1833, that the prolongations mentioned by Dupuytren were not always formed by the palmar fascia; but that they seemed to me to depend on the transformation of the subcutaneous cellular tissue into fibrous bands. M. Goyraud, surgeon to the Hôtel-Dieu of Aix has proved the correctness of this opinion by his researches on permanent contraction of the finger, published in the third volume of the Memoirs of the Royal Academy of Medicine. In addition to a carefully performed dissection, M. Goyraud relates the following case, which confirms my view of the nature of this disease.

"M. Chaine, steward of the hospital, presents a remarkable example of the permanent contraction of the fingers. The three last fingers of the right hand are flexed; of the left, the four last are in the same condition. The left ring-finger is more flexed than the others; on the right side, the little finger is the one most contracted; the middle fingers are only semi-flexed. The contraction came on gradually, and without any pain. M. Chaine is now 58 years of age; when at the age of 42, he first perceived that he was unable to extend the left ring-finger completely. The latter became gradually contracted, and the affection soon extended to the two neighboring fingers. Shortly afterwards, the right little finger began to contract, and then the ring and middle fingers of the same hand. The first phalanges are now flexed at a right angle on the metacarpal bones, and the second phalanges at various angles on the first. On attempting to extend the fingers, it is evident that they are retained in the flexed position by a number of bands running from the palmar fascia to the middle part of the fingers."

In this case, the disease consists in flexion of the first phalanx on the metacarpal bone, and of the second phalanx on the first; the joints of the third phalanges are completely free.

Hence, since contraction of the palmar fascia and its digital prolongations could only influence the first phalanx, it is evident that the contraction of the second phalanx must depend on some other cause. Even in the lectures of Dupuytren we may find a proof of this. Mr. L. had contraction of the ring and little fingers of the left hand, which were completely flexed on the palm; the second phalanx was bent on the first, and the tip of the third applied to the ulnar edge of the palm; the little finger was constantly flexed on the palm of the hand also. M. Dupuytren operated in the following manner:—He commenced by making a transverse incision, ten lines in length, opposite the metacarpo-phalangeal joint of the ring-finger, the knife divided first the skin, and then the palmar fascia. The ring-finger was now reduced nearly to its natural position.

Being desirous of sparing the pain of a second incision, Dupuytren endeavored to prolong the incision of the palmar fascia, towards the ulnar edge of the hand, but could not succeed; he was, therefore, compelled to make a transverse incision opposite the articulation between the first and second phalanx of the little finger, and thus separated its point from the palm, but the remainder of the finger was still immovable. A third incision divided the skin and fascia, opposite the metacarpo-phalangeal joint of the little finger; the benefit was very slight, and he was forced to make an incision opposite the middle of the last phalanx, when the finger at once became straight. Is it not clear, from this case, that other parts besides the palmar fascia were engaged in the disease? Do not we all know that the prolongations of this fascia are attached to the sides and not to the middle of the first phalanx?

From the facts which have just been mentioned, M. Goyraud concludes that permanent contraction of the fingers never depends on the palmar fascia; that we should avoid dividing this latter in any operation for the relief of the disease, and that Dupuytren, probably, divided the subcutaneous bands developed in the cellular tissue, and not prolongations from the palmar aponeurosis.

This opinion seems to me to be too exclusive, and I agree with M. Sanson in thinking that permanent contraction of the fingers may depend on the palmar fascia, on the skin, or more frequently on transformation of the cellular tissue into fibrous bands.

The treatment of this affection is exclusively surgical; we can expect no benefit from any other means than division of the fibrous bands by which the finger is retained in the flexed position. Dupuytren made an assistant extend the finger as far as possible, and then divided the most tense part of the band; if the finger could now be straightened completely, the operation was finished; if not, he made one or more incisions above or below the first one. When the fingers became free, he maintained them in the extended position by means of bandages and splints, for a month or six weeks; but as soon as the state of the wound permitted, he commenced movements of flexion and extension.

M. Goyraud operates in a different way: he makes his incision along the side of the band, and in the same direction with it, and then divides the band in various places, or excises a portion of it. This method has many advantages over that of Dupuytren; the patient runs less risk of inflammation extending along the sheaths of the tendons, or palm of the hand; it allows us to commence moving the fingers at an earlier period, and leaves a much smaller cicatrix. When we have restored the finger to its natural position by means of operation, we are not to think that the patient is free from the danger of relapse. Considerable care must be bestowed on the after-treatment; the joints must be frequently moved; discutient lotions, &c., applied, and the patients must be particularly cautioned against using any hard bodies, or employing himself in any occupation by which the palm of the hand may be irritated.—*Prov. Med. and Surg. Journ.*, Oct. 1841.

